# Murine Norovirus 1 (MNV1) Replication Induces Translational Control of the Host by Regulating eIF4E Activity during Infection*

**DOI:** 10.1074/jbc.M114.602649

**Published:** 2015-01-05

**Authors:** Elizabeth Royall, Nicole Doyle, Azimah Abdul-Wahab, Ed Emmott, Simon J. Morley, Ian Goodfellow, Lisa O. Roberts, Nicolas Locker

**Affiliations:** From the ‡ University of Surrey, Faculty of Health and Medical Sciences, School of Biosciences and Medicine, Guildford GU2 7HX, United Kingdom,; the § Division of Virology, Department of Pathology, University of Cambridge, Addenbrooke's Hospital, Hills Road, Cambridge CB2 2QQ, United Kingdom, and; the ¶ Department of Biochemistry and Molecular Biology, School of Life Sciences, University of Sussex, JMS Building, Brighton BN1 9RH, United Kingdom

**Keywords:** Eukaryotic Translation Initiation Factor 4E (eIF4E), Host-Pathogen Interaction, Mitogen-activated Protein Kinase (MAPK), Protein Synthesis, RNA Virus, Translation Control

## Abstract

Protein synthesis is a tightly controlled process responding to several stimuli, including viral infection. As obligate intracellular parasites, viruses depend on the translation machinery of the host and can manipulate it by affecting the availability and function of specific eukaryotic initiation factors (eIFs). Human norovirus is a member of the Caliciviridae family and is responsible for gastroenteritis outbreaks. Previous studies on feline calicivirus and murine norovirus 1 (MNV1) demonstrated that the viral protein, genome-linked (VPg), acts to direct translation by hijacking the host protein synthesis machinery. Here we report that MNV1 infection modulates the MAPK pathway to activate eIF4E phosphorylation. Our results show that the activation of p38 and Mnk during MNV1 infection is important for MNV1 replication. Furthermore, phosphorylated eIF4E relocates to the polysomes, and this contributes to changes in the translational state of specific host mRNAs. We propose that global translational control of the host by eIF4E phosphorylation is a key component of the host-pathogen interaction.

## Teaser

**Background:** The phosphorylation of eIF4E plays a critical role in controlling protein translation.

**Results:** MNV1 infection results in activation of p-eIF4E, its relocation to polysomes, and translational regulation.

**Conclusion:** MNV1 manipulates the host cell translation machinery by controlling eIF4E activity.

**Significance:** Regulation of cellular response to infection may contribute to viral pathogenesis and persistence.

## Introduction

Human norovirus is the major cause of non-bacterial gastroenteritis in the developed world (1–3). Worldwide, noroviruses are responsible for an estimated 218,000 deaths per annum in children under the age of 5 years and 1.1 million hospital admissions, with outbreaks often occurring in closed facilities, such as hospitals (4). It is estimated that norovirus infection results in a loss of £110 million to the United Kingdom National Health Service every year due to more than 45,000 hospital bed closures (5). The genogroup GII genotype 4 (GII.4) strains are responsible for the majority of human norovirus outbreaks, including pandemics, and were responsible for over one million cases in 2012–2013 in the United Kingdom alone (6). Although norovirus infection mainly results in acute and self-resolving symptoms, it can also contribute to inflammatory bowel disease or neonatal enterocolitis (7–9) and has been reported to cause persistent infections in the immunocompromised and the elderly (10, 11). The *Norovirus* genus is a member of the Caliciviridae family of viruses, having a small single-stranded positive-sense RNA genome. Members of the Caliciviridae typically have genomes ranging from 7.3 to 8.3 kb in length that are polyadenylated at the 3′ end but, unlike eukaryotic mRNAs, have a viral protein, genome-linked (VPg),^4^ covalently attached at the 5′ end. This raised questions about the molecular mechanisms for translation of the viral RNA (12). A detailed understanding of the replication cycle and pathogenesis of human noroviruses is limited due to the lack of an efficient cell culture system to propagate the virus (13, 14). However, the related caliciviruses murine norovirus (MNV1) and feline calicivirus (FCV) can be propagated in cell culture and are used as models that have helped to dissect the norovirus life cycle (15–17).

Viruses are obligate intracellular parasites and depend on the host translational machinery to produce viral proteins. Viral mRNAs have therefore evolved mechanisms to enable them to compete with the host mRNAs for cellular ribosomes and translation factors. Moreover, translational control enables the cell to adjust rapidly to its environment by regulating the translation rate of selected mRNAs and therefore provides an ideal strategy for delivering the targeted responses required during viral infection. Generally, translational control is exerted at the initiation stage, during which ribosomes are recruited to the 5′ end of the cellular mRNA typically bearing a cap structure 7^Me^GpppN (18). The interaction between the ribosome and the mRNA is facilitated by the eukaryotic initiation factor 4F (eIF4F) complex consisting of eIF4E; the cap binding protein, eIF4G; a scaffolding protein; and eIF4A, an RNA helicase required to unwind RNA structure during ribosome scanning. Once eIF4F is bound to the cap, it acts as a point of attachment for ribosomes, which then scan the messenger RNA to locate the AUG start codon and initiate translation (18).

Viruses can modulate translation by altering the function of eIFs. For example, this may be achieved by site-specific cleavage of eIF4G or the phosphorylation of eIF2α (19, 20). Among the translation factors, eIF4E is thought to be limiting for translation; thus, regulating the activity of eIF4E is critical for cellular function (21). The activity of eIF4E is regulated by the eIF4E-binding proteins (4E-BPs), which inhibit translation initiation by competing with eIF4G for a common binding site on eIF4E (22). The interaction of 4E-BP with eIF4E is prevented when 4E-BP is phosphorylated by mTOR, a downstream kinase within the phosphatidylinositol 3′ kinase-Akt-mammalian target of rapamycin (PI3K-Akt-mTOR) pathway (23, 24). Viruses can manipulate this pathway to regulate 4E-BP1 phosphorylation and therefore eIF4E availability (20, 25, 26). For example, poliovirus and vesicular stomatitis virus induce the dephosphorylation of 4E-BP1 to limit eIF4E availability and cellular translation (20). The activity of eIF4E is also controlled by its phosphorylation status. The mitogen-activated protein kinase interacting kinases Mnk1/2 phosphorylate Ser-209 of eIF4E (27). The control of eIF4E phosphorylation depends not only on the activation of Mnk1/2 by signaling cascades of the mitogen-associated protein kinases p38 and ERK1/2 (28) but also on the ability of Mnk1/2 to access eIF4E when both eIF4E and Mnk1/2 are bound to eIF4G (29–31). Infections with herpes simplex virus (HSV-1) and human cytomegalovirus both lead to an accumulation of phosphorylated eIF4E, whereas influenza virus, poliovirus, and encephalomyocarditis virus induce eIF4E dephosphorylation (reviewed in Ref. 19). Although the consequence of eIF4E phosphorylation for its affinity for the cap remains the subject of debate, this has been shown to control the translation of specific mRNAs encoding proteins associated with cell proliferation, inflammation, and interferon production (32–34).

The calicivirus RNA lacks the canonical cap structure recognized by eIF4E. However, previous studies have shown that the norovirus VPg proteins act as a “cap substitute” to mediate translation by interacting with initiation factors (35–38). Recently, we have shown that MNV1 VPg directly binds to the eIF4F complex and that this process is mediated by a high affinity interaction between eIF4G and the C-terminal domain of VPg (39). Although MNV1 VPg also interacts with eIF4E, the role of this interaction remains to be discovered. The depletion of eIF4E or the addition of 4E-BP1 has little to no impact on MNV1 VPg-linked RNA translation in rabbit reticulocyte lysates or in cells (38, 39).

Based on these studies, we hypothesized that eIF4E could play an important role during the norovirus life cycle through the modulation of eIF4E phosphorylation mediated by the mitogen-activated protein kinase (MAPK) pathway. To address this possibility, we have investigated the phosphorylation status of eIF4E in cells infected with the only norovirus that can undergo a complete replication cycle in cell culture, namely MNV1. Our results suggest that Mnk1 is responsible for the phosphorylation of eIF4E during MNV1 infection. Furthermore, we show that the activation of Mnk1 by the p38 kinase is important during the viral life cycle because impairment of eIF4E phosphorylation by inhibition of these kinases has a deleterious effect on viral replication. Moreover, using polysomal profile analysis, we show that phosphorylated eIF4E relocates to the polysomes during infection, and we provide evidence that this could induce the translational control of a subset of mRNAs during infection. These results suggest that regulation of eIF4E activity plays a role during MNV1 infection to regulate translation of specific host mRNAs.

## EXPERIMENTAL PROCEDURES

### 

#### 

##### Cells and Viruses

Mouse leukemic monocyte-macrophage (RAW264.7) cells (European Collection of Cell Cultures) were grown in Dulbecco's modified Eagle's medium (DMEM) with 4.5 g/liter d-glucose + l-glutamine + pyruvate (Invitrogen), supplemented with 10% (v/v) FBS and 1% (v/v) penicillin/streptomycin (5000 units ml^−1^ penicillin G sodium; 5000 μg ml^−1^ streptomycin sulfate in 0.85% saline (Invitrogen)) at 37°C in 5 % CO_2_. MNV1 strain CW1, propagated in RAW264.7 cells, was described previously (38). Virus titers were estimated by determination of the 50% tissue culture infectious dose (TCID_50_) units per ml. For a multiplicity of infection (MOI) equal to one, cells were infected with 1 TCID_50_ unit/cell.

##### Infection Time Course Experiments

The day before infection, RAW264.7 cells were seeded in duplicate in 35-mm dishes at a density of ∼3.5 × 10^6^ cells/dish to obtain confluent monolayers. The RAW264.7 cells were infected with MNV1 at an MOI of 10 TCID_50_ per cell. Matched cell-free lysates were used for control mock infections. The cells were incubated with 5% carbon dioxide at 37 °C. Two-point time courses for MNV infection were harvested at either 2 and 12 or 6 and 14 hpi. The cells were washed twice with 1 ml of PBS before harvesting the cells in 100 μl of NLB buffer (50 mm HEPES, pH 7.4, 150 mm NaCl, 2 mm EDTA, 2 mm Na_3_VO_4_, 25 mm disodium β-glycerophosphate, complete protease inhibitor mixture (Roche Applied Science), 0.5% Nonidet P-40), followed by centrifugation for 3 min at 595 × *g* in a benchtop centrifuge (Centurian 1000 series, Centurion Scientific; East Preston, UK).

##### SDS-PAGE and Immunoblotting

Protein concentrations of the cell lysates were determined by a bicinchoninic acid (BCA) assay using the Pierce BCA protein assay kit (Thermo Scientific) as per the manufacturer's instructions. The samples were adjusted to the same concentration of between 20 and 50 μg of protein and made up to 30 μl with NLB and SDS (3×) sample buffer (New England BioLabs, Hitchin, UK). The proteins were then separated by SDS-PAGE (Mini Protean TGX gels; Bio-Rad) and transferred to polyvinylidene fluoride (PVDF) Immobilon-P membrane (Merck Millipore) for subsequent immunoblotting using conventional methods (40). Following incubation with primary antibodies, washes, and incubation with secondary antibodies, the membranes were probed for chemiluminescence using SuperSignal West Pico Chemiluminescent Substrate (Thermo Scientific), and the signal was detected on radiographic film (Fuji RX, Fisher).

##### Phosphoantibody Array

The Proteome Profiler Human Phospho-MAPK Array (R&D Systems, Abingdon, UK) was used to analyze activation of ERK and p38 using 300-μg protein samples from lysates of mock- and MNV1-infected RAW264.7 cells isolated at 2 and 12 hpi according to the manufacturer's instructions. The signal was detected on radiographic film (Fuji RX) and quantified using ImageJ software (National Institutes of Health).

##### Antibodies and Chemical Inhibitors

Antibodies against eIF4E and MNV1 NS7 (41, 42) have been described previously. Phosphospecific antibodies to eIF4E (Ser-209), 4E-BP1 (Thr-36/47, Ser-65, and Thr-70), eIF2α (Ser-52), Mnk1 (Thr-197/202), and corresponding total antibodies were obtained from Cell Signaling Technology. Antibody to GAPDH was obtained from Ambion Invitrogen. Secondary antibodies included anti-rabbit HRP and anti-mouse HRP (Dako, Cytomation (Ely, UK)). All antibodies were used in accordance with the manufacturer's instructions. The following chemical inhibitors were used for pathway analysis: SB203580 (Calbiochem, Merck Millipore) targeting p38α/β, SCH772984 (Calbiochem) targeting ERK1/2, and CGP57380 (Tocris bioscience; Abingdon, UK) targeting Mnk1. The inhibitor of the eIF4E-eIF4G interaction, 4E2RCat, was kindly provided by Prof. Jerry Pelletier (McGill University, Montreal, Canada). Sodium arsenite was used to induce eIF2α phosphorylation (30 min, 0.5 mm). The CellTiter-Glo® Luminescent Cell Viability Assay (Promega, Southampton, UK) was used to monitor cell viability over the range of concentrations used for each inhibitor according to the manufacturer's instructions.

##### Signaling Pathway Inhibition

The effect of signaling inhibitors on viral replication was determined by treating confluent monolayers of cells in 35-mm dishes with increasing concentrations of SCH772984 (ERK1/2; 1–20 μm), CGP57380 (Mnk1; 1–20 μm), and SB203580 (p38; 1–25 μm) and incubating them for 1 h at 37 °C with 5% CO_2_ as before. To measure the effect of inhibitors on eIF4E phosphorylation, cells were stimulated with LPS at a concentration of 10 ng μl^−1^, harvested as before at 2 and 12 h after stimulation, and analyzed by Western blot. To measure the effect of inhibitors on viral replication, the cells were infected at an MOI of 0.3 TCID_50_/cell with MNV1 for 1 h at room temperature. Following replacement of the virus-containing medium with fresh medium and inhibitor, the cells were incubated for a further 12 h at 37 °C with 5% CO_2_. Culture supernatants were retained, and virus titer was estimated by determination of TCID_50_ as before.

##### Polysome Profile Analysis

Separation of polysomes by sucrose density gradient ultracentrifugation was carried out as described previously (43). RAW264.7 cells (1–2 × 10^7^), mock- or MNV1-infected (MOI of 6.7 TCID_50_/cell) for 8 h, were incubated with 10 μg/ml cycloheximide for 10 min at 37 °C and 5% CO_2_. Cells were washed on ice with PBS containing 10 μg/ml cycloheximide, harvested by scraping, and spun at 450 × *g* for 5 min at 4 °C. The cell pellet was resuspended in 500 μl of lysis buffer (20 mm Tris, pH 7.5, 300 mm sodium chloride, 15 mm magnesium chloride, 100 μg ml^−1^ cycloheximide, and 1% (v/v) Triton X-100), and the resulting lysates were clarified by centrifugation at 19,000 × *g* for 5 min at 4 °C, frozen in liquid nitrogen, and stored at −80 °C. To separate polysomes, samples were layered onto a 10–50% sucrose gradient in lysis buffer and centrifuged in an SW40Ti rotor (Beckman Coulter, High Wycombe, UK) at 38,000 rpm for 2 h. Gradients were fractionated into 1-ml fractions using a FoxyR1 collection system (Teledyne ISCO, Lincoln NE), and UV absorbance was monitored at 254 nm. To induce run-off of polysomes, cycloheximide was omitted from the lysis and gradient buffers and replaced with 10 mm EDTA. Total RNA was extracted from cell lysates or polysomal fractions using the ZR RNA MiniPrep kit (Zymo Research, Irvine, CA) and analyzed by agarose gel electrophoresis (1%, 1× TBE). To monitor the localization of p-eIF4E in polysomal fractions, fractions 6–10 from each gradient were pooled and analyzed by immunoblotting against eIF4E and p-eIF4E.

##### RNA Isolation, PCR, and Quantitative Real-time RT-PCR

Following polysomal fraction separation, each fraction was first added to an exogenous RNA reference, 50 ng of LysA mRNA from *Bacillus subtillis*, as described before (44). Pooled polysomal fractions (typical fractions 5–9) or non-polysomal fractions were first concentrated using Amicon Ultra-4 centrifugal filter devices (YM-30; Millipore). Total RNA was extracted using ZR Miniprep RNA extraction columns following the manufacturer's instructions and including an on-column TURBO DNase I digestion. Using RNA purified from the total RNA or polysome fractions, first strand cDNA synthesis was performed using equivalent amounts of starting RNA from all samples (first strand cDNA synthesis kit, Roche Applied Science). For PCR analysis, the cDNA was analyzed using the PCR Master Mix (Promega), and the PCR cycle conditions used were 95 °C for 5 min, followed by 30 cycles of 95 °C for 30 s, 55 °C for 30 s, and 72 °C for 1 min. The primer pairs used are described in Table 1. For qPCR analysis, the cDNA was analyzed with the MESA BLUE qPCR MasterMix Plus for SYBR (Eurogentec, Southampton, UK) using a Stratagene MX3005 (Stratagene, La Jolla, CA). All samples were prepared in triplicate. The PCR cycle conditions used were 95 °C for 5 min, followed by 40 cycles of 95 °C for 15 s and 60 °C for 1 min, and the *Ct* values were determined using the MxPro software (Stratagene). The translational state (TS) change was calculated after the abundance of each mRNA in monosomes and polysomes was normalized with respect to the abundance of the added-in control, and then the changes in polysomes were normalized to the changes in monosomes for a given mRNA, and the values were expressed as a function of the TS obtained for mock cells and set to 1.

**TABLE 1:** **Primer pairs used for PCR or qPCR amplification**

Name of gene	Primer (5′–3′)
***4EBP1***	
Forward	TAGCCCTACCAGCGATGAGCCT
Reverse	GTATCAACAGAGGCACAAGGAGGTAT

***18S***	
Forward	AGTCCCTGCCCTTTGTACACA
Reverse	GATCCGAGGGCCTCACTAAAC

***GAPDH***	
Forward	TTCAACGGCACAGTCAAGG
Reverse	CTCAGCACCGGCCTCACC

***mTOR***	
Forward	GCAATAAGCGGTCCCGGACAA
Reverse	GCTTTCTTATGGGCTGGTTCTCCAA

***eIF4B***	
Forward	ATGCGTGGGTGAAGCGAAGCTCT
Reverse	GCTCAGGCGCAGATCTGGAGTC

***Nfkbia***	
Forward	GACGCAGACCTGCACACCCC
Reverse	TGGAGGGCTGTCCGGCCATT

***SRp20***	
Forward	TGAGGATCCCCGAGATGCT
Reverse	CTTACACGGCAGCCACACAGT

***rpS19***	
Forward	CAGCACGGCACCTGTACCT
Reverse	GCTGGGTCTGACACCGTTTC

***rpL32***	
Forward	CACCAGTCAGACCGATATGTGAAAA
Reverse	TGTTGTCAATGCCTCTGGGTTT

***Casp4***	
Forward	CTCTGAGGCTCTTTCCAACG
Reverse	TTCCAACACCTTAAGTGGCTTT

***Cdk9***	
Forward	TGCAAGGGCAGCATCTATC
Reverse	TCATGTCCCTGTGCAGGAT

***MNV1***	
Forward	CACGCCACCGATCTGTTCTG
Reverse	GCGCTGCGCCATCACTC

## RESULTS

### 

#### 

##### eIF4E Phosphorylation Is Important for MNV1 Translation and Replication

**FIGURE 1.**
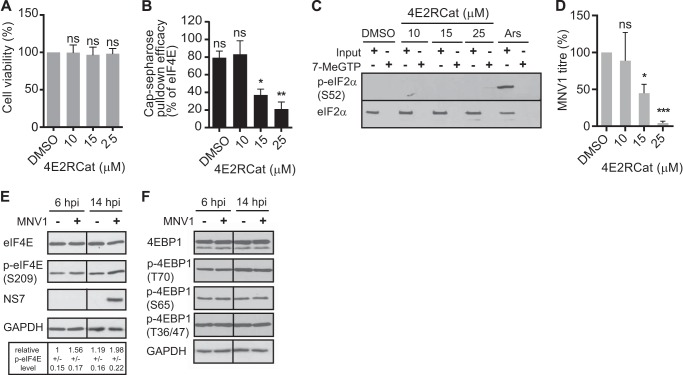
**The eIF4E-eIF4G interaction is important for calicivirus replication.** *A*, cell viability in the presence of 4E2Rcat, an inhibitor of the eIF4E-eIF4G interaction, was determined using CellTiter-Glo® luminescent cell viability assay (Promega). Viability is expressed as a percentage of the DMSO control set to 100%. Results show the means ± S.E. (*error bars*) from three separate experiments. *B*, the efficacy of cap Sepharose pull-down in the presence of 4E2Rcat was determined using immunoblotting for eIF4E and eIF4G. The efficacy of eIF4G pull-down is expressed as a percentage of the DMSO control set to 100% and normalized to the eIF4E pull-down efficacy. Experiments were analyzed by one-way analysis of variance with Dunnett's multiple comparisons test: *, *p* < 0.05; **, *p* < 0.01; ***, *p* < 0.001; *ns*, not significant (GraphPad Prism version 6; GraphPad Software, San Diego, CA). *C*, RAW264.7 cells were pretreated for 1 h with 10–25 μm 4E2RCat or 30 min with 0.5 mm sodium arsenite (*Ars*). Cell lysates were analyzed by immunoblotting, and results shown are representative of three independent experiments. *D*, RAW264.7 cells were pretreated for 1 h with 10–25 μm 4E2RCat. The RAW264.7 cells were subsequently infected at an MOI of 0.3 TCID_50_/cell with MNV1. The figure shows the results of three separate experiments analyzed by one-way analysis of variance with Dunnett's multiple comparisons test: *, *p* < 0.05; **, *p* < 0.01; ***, *p* < 0.001; *ns*, not significant (GraphPad Prism version 6). *E*, RAW264.7 cells were infected with MNV1 at an MOI of 10 TCID_50_/cell and harvested at 6 and 14 hpi. Cell lysates were analyzed by immunoblotting. The infection was monitored by visualization of the viral non-structural protein-7 (NS7), the 3D polymerase. GAPDH was used as a loading control. The results were analyzed by ImageJ software (National Institutes of Health), and levels of eIF4E phosphorylation relative to uninfected cells at 6 hpi are shown ± S.E. *F*, RAW264.7 cells were infected with MNV1 at an MOI of 10 TCID_50_/cell and harvested at 6 and 14 hpi. Cell lysates were analyzed by immunoblotting. GAPDH was used as a loading control.

Previous studies have demonstrated that the VPg protein of caliciviruses acts as a proteinaceous cap substitute to initiate translation by interacting with eIF4E both *in vitro* and *in vivo* (37, 38). However, although the interaction with eIF4E is required for the translation of FCV RNA in *in vitro* RRL systems, it plays little to no role in MNV translation *in vitro* or in murine microglial cells (38, 39). Here we hypothesize that instead of a direct role in viral translation, eIF4E and/or the regulation of its activity might be important during MNV1 replication. 4E2RCat has been identified by high-throughput screening as an eIF4E-eIF4G inhibitor with an IC_50_ of 13.5 μm. It blocks cap-dependent translation *in vitro* and in cells and inhibits coronavirus replication (45, 46). Therefore, to determine whether there exists any requirement for the eIF4E-eIF4G interaction during calicivirus replication, we investigated the effect of 4E2RCat on MNV1 replication. First we ascertained that 4E2RCat did not affect the viability of the host cells. At the concentrations used in our studies, from 10 to 25 μm, no effect was detected on RAW264.7 cell viability (Fig. 1*A*). In addition, we used cap-Sepharose pull-down and immunoblotting as described previously (37, 38) to show that increasing concentrations of 4E2RCat led to a reduction in the amount of eIF4G pulled down relative to eIF4E (Fig. 1*B*). A different inhibitor of the eIF4E-eIF4G interaction, namely 4EGI-1, has been shown to induce eIF2α phosphorylation, leading to decreased translation independently from its effect on eIF4E (47). We therefore verified, by immunoblotting, that 4E2RCat did not induce eIF2α phosphorylation at the concentration used (Fig. 1*C*). We next measured the effect of 4E2RCat on MNV1 replication by infecting RAW264.7 cells at an MOI of 0.3 TCID_50_/cell with MNV1 for 12 h and determining viral titer using a TCID_50_ assay. In the presence of 4E2RCat, the replication of MNV1 was inhibited in a dose-dependent manner with significant inhibition of MNV replication at both 15 and 25 μm concentrations (Fig. 1*D*). This suggests that impairing the interaction of eIF4E with eIF4G inhibits some aspect of the MNV1 life cycle.

In addition to driving the formation of the eIF4F complex and translation, the formation of a complex between eIF4E and eIF4G is also important for the regulation of eIF4E phosphorylation (31, 48). In response to various stimuli acting through signaling cascades of the mitogen-associated protein kinases p38 and ERK, the mitogen-activated protein kinase interacting kinases, Mnk1/2, phosphorylate eIF4E on Ser-209 (27). Importantly, Mnk1/2-mediated phosphorylation of eIF4E occurs only when eIF4E is complexed with eIF4G because Mnk1/2 lack the ability to bind eIF4E directly (48). Several viral infections induce an accumulation of phosphorylated eIF4E that correlates with the inhibition of cellular protein synthesis, whereas other infections can lead to eIF4E dephosphorylation (20). Thus, after showing that perturbation of the interaction between eIF4E and eIF4G inhibits MNV1 replication, we investigated the phosphorylation status of eIF4E during MNV1 infection. First, we evaluated the steady-state levels of eIF4E in cells infected with MNV1 by immunoblotting (Fig. 1*E*). The lysates from mock- and MNV1-infected RAW cells were fractionated by SDS-PAGE and analyzed by immunoblotting at the 6 and 14 hpi time points of the 18-h replication cycle. The MNV1 infection is indicated by the presence of the viral NS7 protein. Whereas there was no obvious change in the level of eIF4E during infection, the level of p-eIF4E increased (Fig. 1*E*). At 6 and 14 hpi, the relative level of p-eIF4E increased from 1 to 1.56 and 1.98 when compared with the mock infection (Fig. 1*E*). Therefore, MNV1 induces the phosphorylation of eIF4E. Furthermore, this does not reflect a general, nonspecific activation of intracellular signaling. Indeed, we could not detect any activation of 4E-BP1 phosphorylation, which has been shown before to be a response to several viral infections (Fig. 1*F*).

##### eIF4E Phosphorylation via MAPK Signaling Pathways Is Important for MNV1 Replication

**FIGURE 2.**
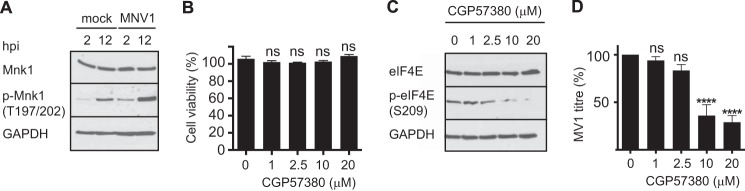
**eIF4E phosphorylation by Mnk1 is important for MNV1 replication.** *A*, RAW264.7 cells were infected with MNV1 at an MOI of 0.3 TCID_50_/cell or mock-infected, and lysates were harvested at 2 and 12 hpi and analyzed by SDS-PAGE and Western blotting. *B*, RAW264.7 cells were treated with CGP57380, a specific Mnk1 inhibitor, at the indicated concentrations, and cell viability was measured using the CellTiter-Glo® luminescent cell viability assay (Promega). *C*, similarly treated RAW264.7 cells were analyzed by SDS-PAGE and Western blot for eIF4E. *D*, RAW264.7 cells were subsequently treated with CGP57380 for 1 h before being infected with MNV1 at a MOI of 0.3 TCID_50_ per cell. The cells were incubated at 37 °C with 5% CO_2_ for 12 h, and the viral titer was estimated by a TCID_50_ assay. *Error bars*, S.E.

The MAPK pathway is responsible for phosphorylating eIF4E in response to external stimuli. The cellular kinases Mnk1/2 phosphorylate eIF4E on Ser-209, and whereas Mnk2 is constitutively activated, Mnk1 is activated by either of the kinases p38 or ERK1/2, via MEK3/6 and MEK1/2, respectively (27, 29, 49). To analyze the importance of eIF4E phosphorylation for MNV1 replication, we monitored Mnk1 activation during MNV1 infection. To this end, the lysates from mock- and MNV1-infected RAW264.7 cells, at 2 or 12 hpi, were fractionated by SDS-PAGE and analyzed by immmunoblotting. The steady-state levels of Mnk1 in cells at 2 or 12 hpi were not affected by MNV1 infection (Fig. 2*A*). However, MNV1 infection led to an increase in Mnk1 phosphorylation (Fig. 2*A*). To further investigate the role of Mnk1 in eIF4E phosphorylation and its importance for MNV1 replication, we analyzed the effect of inhibiting Mnk1 function. RAW264.7 cells were treated with 1–20 μm CGP57380, a specific inhibitor of Mnk1. At these concentrations, this compound had no effect on cell viability while impairing eIF4E phosphorylation (Fig. 2, *B* and *C*). We treated RAW264.7 cells with CGP57380 and infected them for 12 h with MNV1 at an MOI of 0.3 TCID_50_/cell, and the virus titer was determined using the TCID_50_ assay. The addition of 10 or 20 μm CGP57380 impaired MNV1 replication, significantly reducing viral titer by 60 and 67%, respectively (Fig. 2*D*). This suggested that Mnk1 could play a role during MNV1 infection by phosphorylating eIF4E.

**FIGURE 3.**
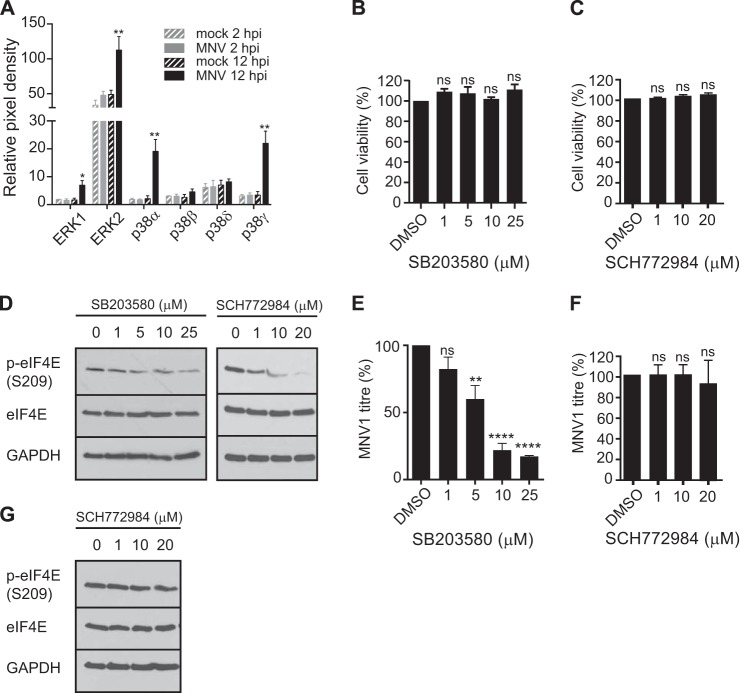
**MAPK signaling through p38 is important for MNV1 replication.** *A*, the Proteome Profiler human phospho-MAPK array (R&D Systems) was used to compare relative quantities of phosphorylated ERK and p38 between 2 and 12 hpi. The arrays were visualized by immunoblotting, and the results were analyzed using ImageJ software. The spots were normalized to control spots on the arrays, and values for relative phosphorylation (activation) of the ERK and p38 isoforms are subsequently presented as relative pixel density. Viability of cells treated with SB203580 (p38) (*B*) and SCH772984 (ERK) (*C*) inhibitors was measured using the CellTiter-Glo® luminescent cell viability assay (Promega). Viability is expressed as a percentage of the DMSO control set to 100%. Results show the means ± S.E. (*error bars*) from three separate experiments. *D*, RAW264.7 cells were treated with SB203580 (1–25 μm) or SCH772984 (1–20 μm) for 1 h as indicated before stimulating with LPS (10 ng/ml). Cell lysates were analyzed by SDS-PAGE and Western blot. *E*, RAW264.7 cells were treated with the same range of concentrations of inhibitor as used for the viability assay with SB203580 and then incubated at 37 °C with 5% CO_2_ for 1 h before infection with MNV1 at an MOI of 0.3 TCID_50_/cell. Cells were harvested at 12 hpi, and the virus titer was estimated by TCID_50_. *F*, RAW264.7 cells were treated similarly with SCH772984 inhibitor at the indicated concentrations and infected with MNV1, and again MNV1 titer was estimated by determination of TCID_50_ at 12 hpi. The figure shows the results of three separate experiments analyzed by one-way analysis of variance with Dunnett's multiple comparisons test: *, *p* < 0.05; **, *p* < 0.01; ***, *p* < 0.001; *ns*, not significant (GraphPad Prism version 6). *G*, RAW264.7 cells were treated similarly with SCH772984 inhibitor at the indicated concentrations and infected with MNV1, and cell lysates were analyzed by SDS-PAGE and Western blot.

The MAP kinases p38 and ERK1/2 can activate Mnk1 to regulate the phosphorylation of eIF4E. Therefore, to consolidate our hypothesis that eIF4E phosphorylation contributes to the MNV1 life cycle, we used an MAPK antibody array to examine the activation of ERK1/2 and p38. Using the lysates of mock- and MNV1-infected RAW264.7 cells, we monitored the phosphorylation of ERK1 and ERK2 and the different isoforms of p38 at 2 and 12 hpi. These data, summarized in Fig. 3*A*, show that at both 2 and 12 hpi, ERK1 and ERK2 are activated during MNV1 infection. In addition, the main p38 isoform, p38α, shows strong activation during MNV1 infection and similarly for p38γ, whereas p38β and p38δ showed no significant change. To further dissect whether the activation of p38 and ERK1/2 may be required for MNV1 replication, we used chemical inhibitors of p38 and ERK1/2 kinase activity, SB203580 and SCH772984, respectively. First, we ensured that at the concentrations tested, the inhibitors did not affect cell viability (Fig. 3, *B* and *C*) and that they reduced eIF4E phosphorylation (Fig. 3*D*). RAW264.7 cells were then treated with SB203580 or SCH772984 and infected at an MOI of 0.3 TCID_50_/cell with MNV1 for 12 h, and the virus titer was estimated by determination of TCID_50_. The addition of SB203580 resulted in a dose-dependent reduction of eIF4E phosphorylation and MNV1 titer, with an 82% inhibition of replication at 25 μm (Fig. 3, *D* and *E*). The addition of SCH772984 also impaired the phosphorylation of eIF4E (Fig. 3*D*). However, we could not detect any significant effect on MNV1 replication upon ERK1/2 inhibition (Fig. 3*F*). This might be due to the differential effect of p38 and ERK on Mnk1 activation. Indeed, whereas ERK1/2 can activate both Mnk2, responsible for constitutive eIF4E phosphorylation, and Mnk1, responsible for inducible eIF4E phosphorylation, p38 selectively targets Mnk1 (50). These results demonstrate that MNV1 infection triggers the activation of Mnk1 and that although both upstream kinases ERK and p38 are activated, only the activation of p38 is required for MNV1 replication. To support this, RAW264.7 cells were then treated with SCH772984 and infected at an MOI of 0.3 TCID_50_/cell with MNV1 for 12 h, and the phosphorylation of eIF4E was monitored by immunoblotting. The addition of SCH772984 did prevent eIF4E phosphorylation during infection (Fig. 3*G*). These data further support a role for eIF4E phosphorylation during infection, driven by p38 rather than ERK.

##### p-eIF4E Relocates to Polysomes during MNV1 Infection

**FIGURE 4.**
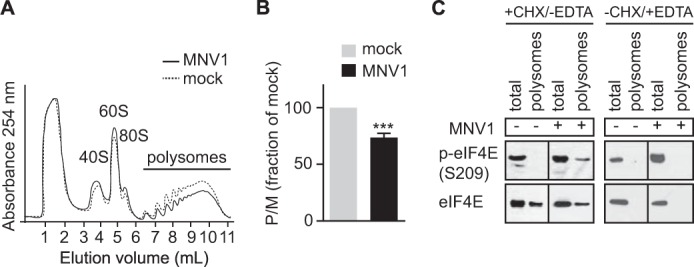
**Polyribosome analysis of mock- or MNV1-infected cells.** *A*, lysates from mock-infected or MNV1-infected (MOI of 6.7 TCID_50_/cell) RAW264.7 cells were prepared as detailed under “Experimental Procedures” following 8 h of infection. A normalized (by *A*_254_) amount of lysate was separated on a 10–50% sucrose gradient and fractionated into 1-ml fractions using a FoxyR1 collection system (Teledyne). The displayed trace represents absorbance at 254 nm (*vertical axis*) throughout the gradient from top (*left*) to bottom (*right*). 40 S (small ribosomal subunit), 60 S (large ribosomal subunit), 80 S (monosome), and polysome peaks are *labeled. B*, the areas *below* the monosome and polysome peaks were determined for several biological replicates (*n* = 3), and the mean polysome/monosome (*P/M*) ratio was calculated and normalized (as a percentage of the mock-infected). The ratio of the area under the polysomal (*P*) to monosomal (*M*) peaks is shown (*P/M*), calculated using standard area under the curve methods. *C*, analysis by immunoblotting of the protein recovered from the profiling assay shows that phosphorylated eIF4E is detected only in the polysomal fractions (fractions 6–10 from the gradient) from MNV1-infected lysates. Polysome run-off was induced as indicated using EDTA. Results are representative of three separate experiments. *Error bars*, S.E.

**FIGURE 5.**
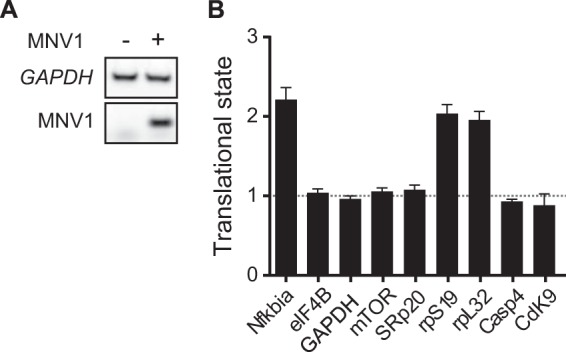
**Analysis of changes in TS during MNV1 infection.** *A*, semiquantitative RT-PCR analysis of GAPDH and MNV1 mRNA among total RNA isolated from the polysomal fractions of mock- or MNV1-infected cells. Total RNAs were isolated from the pooled translationally active polysomal fraction and inactive free fraction of mock- or MNV1-infected cell lysates and subjected to reverse transcription using the Transcriptor first strand cDNA synthesis kit (Roche Applied Science). PCR amplification was carried out using PCR Master Mix (Promega). *B*, status of polysomal and nonpolysomal abundances of mRNAs upon MNV1 infection. Total RNAs were isolated from the translationally active polysomal fraction and inactive free fraction of mock- or MNV1-infected cell lysates and subjected to reverse transcription using a Transcriptor first strand cDNA synthesis kit (Roche Applied Science). PCR amplification was carried out using MESA BLUE qPCR MasterMix Plus for SYBR (Eurogentec) and an Mx3005 qPCR system (Stratagene). Shown are qPCR results from three independent biological isolates of both mock- and MNV1-infected cells. The *bars* represent the mean, and the *error bars* show the S.E. The change in TS has been calculated using the formula, (mock/MNV1)_poly_/(mock/MNV1)_mono_, where (mock/MNV1)_poly_ and (mock/MNV1)_mono_ represent the changes of individual mRNAs in polysomal and monosomal RNA, respectively.

It has been shown that p-eIF4E is involved in the translational control of specific mRNAs involved in cell survival and inflammation (32, 51, 52). Moreover, a recent study demonstrated that p-eIF4E regulates interferon production by controlling the translation of *Nfkbia* mRNA, thereby modulating sensitivity to viral infections (33). Thus, we hypothesized that p-eIF4E could play a role during MNV1 infection by altering the translation of a subset of mRNAs. Therefore, we used polysome analysis to isolate the mRNAs associated with translationally active ribosomes in MNV1-infected cells. First, cell lysates from mock- or MNV1-infected cells at 8 hpi (representing an early time during infection with ongoing RNA and protein synthesis) were prepared and subjected to a 10–50% sucrose gradient centrifugation. A typical polysome profile pattern was obtained, as shown in Fig. 4*A*. In contrast to the mock-infected cells, MNV1-infected cells exhibited a moderate translational defect as shown by a decrease in the amount of polysomes (Fig. 4*A*). This was further quantified by monitoring the ratio of polysomes to monosomes. Quantification of the areas under the monosome and polysome peaks shows that the polysome/monosome (*P*/*M*) ratio of MNV1-infected cells is 74% that of mock-infected cells (Fig. 4*B*). Thus, a fraction of free ribosomal subunits is no longer engaged in mRNA translation during MNV1 infection, which could suggest a moderate general inhibition of mRNA translation. Subsequently, we analyzed the association of p-eIF4E with translationally active polysomes. We observed that the amount of total eIF4E in both total lysates and pooled polysomal fractions was similar in mock- and MNV1-infected cells (Fig. 4*C*). However, we found that p-eIF4E was associated with polysomal fractions from MNV1-infected cells but not mock-infected cells (Fig. 4*C*). The association of p-eIF4E with polysomes was impaired when run-off of polysomes was induced with EDTA, suggesting that p-eIF4E does not sediment with high density complexes in the absence of polysomes (Fig. 4*C*). These results suggest that p-eIF4E relocates to polysomes during MNV infection and support the idea that the phosphorylation of eIF4E contributes to the viral life cycle. To further investigate whether translation of specific mRNAs is altered during MNV1 infection in order to modulate the host-pathogen interactions, we analyzed mRNA translation status using RT-qPCR. To address this, we compared the relative abundance of a set of mRNAs, including the p-eIF4E-sensitive *Nfkbia* mRNA, and other mRNAs previously proposed to be controlled by p-eIF4E (SRp20, CDK9, Casp4, rpS19, and rpL32) in the pooled polysomal and non-polysomal fractions in mock- and MNV-1-infected cells to define their TS (53). First, using RT-PCR, we were able to detect the presence of a control mRNA, GAPDH, in polysomes of both mock- and MNV1-infected cell lysates, whereas MNV1 RNA was only detected in infected lysates (Fig. 5*A*). Then we measured the abundance of target mRNAs, in pooled polysome or non-polysome fractions, using RT-qPCR. The changes in abundance in polysome and non-polysome fractions were normalized to an added-in exogenous RNA control, and the TS represents the relative changes in abundance in polysome fractions normalized to changes in non-polysome fractions for a given mRNA during infection (Fig. 5*B*). Using this analysis, a TS of >1 represents a translational activation of a particular mRNA, whereas a TS of <1 represents translational repression. Our results revealed that during MNV1 infection, *Nfkbia* mRNA is translationally activated (TS = 2.2). This could correlate with the relocation of p-eIF4E to polysome fractions because previous studies have demonstrated that the translational activation of *Nfkbia* mRNA is dependent on p-eIF4E (33). This in turn impairs interferon production by up-regulating the translation of the NF-κB inhibitor IκBα and leads to increased sensitivity to viral infection (33). Therefore, the stimulation of polysome-associated phosphorylated eIF4E might reflect one mechanism by which MNV1 dampens the response of the cell to the infection. Furthermore, rpS19 and rpL32 were also translationally activated (TS = 2.04 and 1.93, respectively). No activation was detected for CDK9, Casp4, and SRp20, which could reflect that p-eIF4E alone is not sufficient for their translational activation and that additional cellular factors might be required. Other mRNAs, such as mTOR, GAPDH, or eIF4B, were unaffected. Therefore, MNV1 infection leads to relocation of p-eIF4E to polysomal fractions and changes in the translational state of specific mRNAs.

## DISCUSSION

Caliciviruses have developed strategies to divert the host cell translation apparatus to their mRNAs using the VPg protein, attached at their 5′ end, which acts as a cap substitute by interacting with eukaryotic initiation factors (35, 37–39, 54). The FCV VPg binds directly to eIF4E to direct translation *in vitro* (37). Although an interaction between the MNV1 VPg and eIF4E has been demonstrated, its relevance for MNV1 translation remained unknown because it can be removed or sequestered *in vitro* with little to no impact on MNV translation (38). We have recently demonstrated that translation of the MNV1 RNA is driven by a high affinity interaction between eIF4G and the C-terminal domain of VPg (39). In addition, the siRNA-mediated reduction of eIF4E expression or its depletion through the overexpression of 4E-BP1 had no effect on MNV1 replication (39). Several viruses manipulate signaling pathways to alter 4E-BP1 phosphorylation and eIF4E availability, whereas eIF4E phosphorylation itself has been shown to be important for the replication of some viruses (20, 55). For example, vesicular stomatitis virus infection results in the dephosphorylation of eIF4E and 4E-BP1, whereas eIF4E phosphorylation enhances HSV-1 replication (56, 57). This prompted us to investigate how calicivirus infection may influence eIF4E activity. We began by investigating the effect on MNV1 replication of 4E2RCat, a molecule that prevents the interaction between eIF4E (the cap-binding protein) and eIF4G (the eIF4F large scaffolding protein) and impairs coronavirus replication (45, 58). We found that disruption of the eIF4E-eIF4G interaction adversely affected the replication of MNV1 (Fig. 1). Therefore, eIF4E, via its interaction with eIF4G, plays a role during MNV1 replication, either directly or indirectly.

It has been proposed previously that Mnk-mediated eIF4E phosphorylation depends not only on the activation of Mnk itself but also on the accessibility of eIF4E through the recruitment of eIF4E to the eIF4G-Mnk complex (29, 31). In fact, restricting Mnk access to eIF4E via the eIF4E inhibitor 4E-binding proteins, 4E-BPs, limits the phosphorylation of eIF4E (31). Therefore, we investigated whether the requirement for the eIF4E-eIF4G interaction was important because it mediates eIF4E phosphorylation during MNV1 infection rather than for any direct role during viral translation. We detected little change in the level of total eIF4E during MNV1 infection, whereas the level of phosphorylated eIF4E increased during MNV1 infection (Fig. 2). By modulating MAPK pathways, viruses can control eIF4E phosphorylation, as seen with reovirus, HSV-1, or HCV (59–61). The MAPK-interacting kinases Mnk-1/2 phosphorylate eIF4E on the conserved physiological site at serine 209 upon their activation by MEK1/2 via ERK1/2 and p38 (62, 63). In agreement with our observation that eIF4E phosphorylation increases during infection, we also detected activation of Mnk1, ERK1/2, and p38 phosphorylation (Figs. 2 and 3). Furthermore, inhibition of the signaling pathways governing phosphorylation of eIF4E using inhibitors of p38 (SB203580) and Mnk1 (CGP57380) caused a significant decrease in the replication of MNV1, whereas the inhibition of ERK1/2 had no effect (Figs. 2 and 3). This implies that although both the p38 and ERK arms of the MAPK pathway contribute to Mnk1/2 and eIF4E phosphorylation during MNV1 infection, only p38 is required for viral replication.

Although the effect of eIF4E phosphorylation on its affinity for the cap remains the subject of research, phosphorylation of serine 209 has been linked with cell survival and proliferation in a number of cancers, including prostate cancer (32, 34, 51, 64, 65). It has been proposed that p-eIF4E can stimulate the translation of specific mRNAs, including mRNAs involved in proliferation and the inhibition of apoptosis but also involved in the control of inflammation and interferon production (32, 33, 52). For example, Mnk1 activation and eIF4E phosphorylation can promote the synthesis of IRF8 and the expression of M1 macrophage-associated genes (52). Furthermore, the loss of eIF4E phosphorylation in cells expressing the eIF4E mutant S209A is associated with impaired translation of the *Nfkbia* mRNA, which encodes the NF-κB inhibitor IκBα; this leads to an enhanced type I IFN response that protects against viral infection (33). Fitting with this observation, viral infections known to cause dephosphorylation of eIF4E (*e.g.* vesicular stomatitis virus) result in a reduced polysomal loading of *Nfkbia* mRNA and activation of NF-κB, which leads to interferon production (33). This highlights a direct involvement of eIF4E phosphorylation and translational control of a subset of mRNAs in the host response to infection. The fractionation of polysomes from MNV1 or mock-infected cells supports a role for p-eIF4E in the translation of specific mRNAs during MNV1 infection. Indeed, whereas the amount of polysomes decreased during infection to 75% (Fig. 4), which could represent a drop in overall translation,^5^ we observed an accumulation of phosphorylated eIF4E in polysomes (Fig. 4). This was not the case for the mock infection, where no such accumulation was observed. Thus, it appears that MNV1 may induce eIF4E phosphorylation to maintain cell proliferation during infection or to control the translation of specific mRNAs involved in the antiviral response. Supporting this hypothesis, we showed that the expression of the *Nfkbia* mRNA, sensitive to eIF4E phosphorylation, was up-regulated during infection (Fig. 5). In addition, other p-eIF4E-sensitive mRNAs, rpS19 and rpL32, were translationally activated, but not all. This would support a model in which additional factors, like RNA-binding proteins or microRNAs, would contribute to the translational activation of p-eIF4E-sensitive mRNAs in a specific biological context.

MNV is an enteric pathogen that is interferon-sensitive and infects macrophages and dendritic cells *in vivo*, sometimes resulting in long term persistent infections that are lifelong and occur despite the presence of an antibody and cellular immune response (66–68). Therefore, it is likely that MNV uses several strategies to avoid or control the cellular response to infection. Type I interferons (IFNα and IFNβ) are widely expressed cytokines that constitute a major component of the innate immune system, acting as the first line of defense against virus infections (69). IFN can elicit distinct and specific upstream signals to modulate translation and is highly sensitive to eIF4E availability (70, 71). The only innate antagonist identified to date, VF1, appears to regulate activation of the innate immune response by antagonizing the induction of IFNβ and delaying apoptosis (72). Like many viruses, it is likely that MNV has evolved several mechanisms to control the host response to infection due to the redundancy of the innate immune response to infection. Fitting with this hypothesis, the NS1/2 and VP2 proteins have recently been shown to contribute to viral persistence (67, 73). Here we describe an additional mechanism by which MNV infection may regulate the response to infection, namely the regulation of eIF4E activity, which is supported by the fact that the phosphorylation of eIF4E negatively regulates interferon production (33). Human astrovirus, another positive-sense single-stranded RNA virus encoding a VPg essential for viral infectivity, also activates ERK1/2, and inhibition of MEK1/2 significantly impairs viral replication (74, 75). Controlling eIF4E phosphorylation could therefore be a more general mechanism that RNA viruses use to modulate translation during infection and control the host response to infection.

Overall, several of our results suggest that p-eIF4E plays a key role during MNV1 replication: MNV replication is impaired following disruption of the eIF4E-eIF4G interaction; blocking the signaling pathways leading to eIF4E phosphorylation inhibits replication; and phosphorylated eIF4E relocates to polysomes during infection. However, the impact of the eIF4E-VPg interaction observed before on these effects remains unclear. Independently of its cap-binding activity, eIF4E exhibits an additional function and stimulates eIF4A helicase activity, which can mediate mRNA restructuring (76). Because eIF4A is required for MNV1 translation, the role of eIF4E recruitment to VPg could be to ensure optimal helicase activity and unwinding of the structured 5′ region of the MNV1 genome, whereas the role of a potential interaction between p-eIF4E and VPg during infection is under investigation (38). To integrate all of these data, we propose that the eIF4E-eIF4G interaction is important to ensure that eIF4E can be efficiently phosphorylated via the ERK and p38 pathways during MNV1 infection because the phosphorylation of eIF4G-bound eIF4E requires the interaction of Mnk1/2 with eIF4G (48). This would therefore explain why both the eIF4E-eIF4G interaction and the activation of the MAPK signaling pathways are required for MNV1 replication, whereas eIF4E does not play a direct role in viral translation. The association of phosphorylated eIF4E with actively translating ribosomes during MNV infection could facilitate the translation of as yet unidentified cellular mRNAs, including *Nfkbia*, to modulate the immune response, as suggested by Herdy *et al.* (33). Our findings support a model in which one of the mechanisms used by caliciviruses to control cellular translation during infection is to modulate the phosphorylation of eIF4E through the MAPK cell signaling pathways to ensure survival within the host.

## Footnotes

5 E. Emmot and I. Goodfellow, manuscript in preparation.

## Footnotes

4 The abbreviations used are:

VPg: viral protein, genome-linked
MNV: murine norovirus
FCV: feline calicivirus
eIF: eukaryotic initiation factor
4E-BP: eIF4E-binding protein
TCID_50_: 50% tissue culture infectious dose
MOI: multiplicity of infection
hpi: hours postinfection
p-eIEF4E: phosphorylated eIF4E
qPCR: quantitative PCR.
